# In Situ Fabrication of N-Doped ZnS/ZnO Composition for Enhanced Visible-Light Photocatalytic H_2_ Evolution Activity

**DOI:** 10.3390/molecules27238544

**Published:** 2022-12-04

**Authors:** Jinhua Xiong, Xuxu Wang, Jinling Wu, Jiaming Han, Zhiyang Lan, Jianming Fan

**Affiliations:** 1 State Key Laboratory of Photocatalysis on Energy and Environment, Fuzhou University, Fuzhou 350002, China; 2 Fujian Provincial Key Laboratory of Clean Energy Materials, Longyan University, Longyan 364000, China

**Keywords:** ZnS/ZnO, N-doped, photocatalytic H_2_ evolution, heterojunction

## Abstract

For achieving the goal of peaking carbon dioxide emissions and achieving carbon neutrality, developing hydrogen energy, the green and clean energy, shows a promising perspective for solving the energy and ecological issues. Herein, firstly, we used the hydrothermal method to synthesize the ZnS(en)_0.5_ as the precursor. Then, ZnS/ZnO composite was obtained by the in situ transformation of ZnS(en)_0.5_ with heat treatment under air atmosphere. The composition, optical property, morphology, and structural properties of the composite were characterized by X-ray photoemission spectroscopy (XPS), Ultraviolet-visible absorption spectra (Uv-vis Abs), Scanning electron microscopy (SEM) and Transmission electron microscopy image (TEM). Moreover, the content of ZnO in ZnS/ZnO was controlled via adjustment of the calcination times. The visible-light response of ZnS/ZnO originated from the in situ doping of N during the transformation of ZnS(en)_0.5_ to ZnS/ZnO under heat treatment, which was verified well by XPS. Photocatalytic hydrogen evolution experiments demonstrated that the sample of ZnS/ZnO-0.5 h with 6.9 wt% of ZnO had the best H_2_ evolution activity (1790 μmol/h/g) under visible light irradiation (λ > 400 nm), about 7.0 and 12.3 times that of the pure ZnS and ZnO, respectively. The enhanced activities of the ZnS/ZnO composites were ascribed to the intimated hetero-interface between components and efficient transfer of photo-generated electrons from ZnS to ZnO.

## 1. Introduction

Hydrogen, as a clear and renewable energy, has been considered as a candidate for future energy use. Photocatalysis is a powerful technology for transforming solar energy into chemical energy [1,2,3]. The search for and design of photocatalysts are the key ways for achieving the highly efficient photocatalytic hydrogen evolution [4,5].

ZnS is one of the well-known photocatalysts for H_2_ production, but suffers from a ultraviolet response, a low surface area and a repressed carriers migration [6]. As reported by our previous work [7], designing ZnS with a porous plate-like structure and N-doping in crystal lattice is a feasible approach to broaden the optical absorption and increase the surface areas of ZnS. Therefore, to further enhance the photocatalytic hydrogen evolution activity of N-doped porous ZnS nanoplates, one’s attentions should be focused on the issues of carriers separation. Over the past decades, engineering heterojunctions in photocatalysts have been proved to be a promising route for promoting carriers separation [8,9]. One of the most vital factors for the effective separation of electron–hole pairs involves the ohmic contact interface and energy band structures of different components in photocatalyst [10,11]. Fortunately, ZnO, as another important II-VI group semiconductor, has similar physical and chemical properties to ZnS [12]. Their energy band structures are generally staggered, forming a so-called type II heterojunction, which facilitates the separation of carriers [13,14]. More importantly, hexagonal ZnS and ZnO are isomorphous compounds and a mutual transformation between ZnS and ZnO can be executed via anion exchange [15]. Hence, the preparation of the composition of ZnS/ZnO by partial sulfuration of ZnO or oxidation of ZnS could form a firm heterojunction with a superior ohmic contact interface [16,17,18] This has been further confirmed by recent reports. As Wang et al. reported [19], an all-solid-state Z-Scheme ZnS-ZnO heterostructure photocatalyst was prepared via in situ sulfurization of ZnO sheets. The obtained ZnS-ZnO showed a remarkable enhancement of photocatalytic H_2_ evolution activity, but pursuing visible-light activity was still in demand. Additionally, Cheng et al. reported a ZnO/ZnS heteronanostructure for photocatalytic H_2_ evolution under visible light (λ >420 nm) [20]. The method for preparing ZnO/ZnS suffered from a tedious process, including synthesis of Zn-MOF, partial sulfuration of Zn-MOF, high temperature treatment for in-situ transformation of ZnS@Zn-MOF into ZnS@C, and further oxidation of ZnS@C in air into ZnS/ZnO. The visible-light photocatalytic H_2_ evolution activity of the ZnS/ZnO was not more desirable. Therefore, engineering a ZnS/ZnO heterojunction photocatalyst with visible-light response for photocatalytic H_2_ evolution is still imperative and challenging. For addressing these terms, this work provides a feasible and simple way to fabricate a ZnS/ZnO photocatalyst for H_2_ evolution with visible-light response and a heterojunction structure simultaneously. 

Herein, N-doped ZnS/ZnO composition was prepared by the in situ transformation of ZnS(en)_0.5_ with heat treatment under air atmosphere. The content of ZnO in ZnS/ZnO was able to be controlled via adjustment of the calcination times. Photocatalytic hydrogen evolution experiments demonstrated that the sample of ZnS/ZnO-0.5 h with 6.9 wt% of ZnO had the best H_2_ evolution activity (1790 μmol/h/g) under visible light irradiation (λ > 400 nm), about 7.0 and 12.3 times that of the pure ZnS and ZnO, respectively. The enhanced activities of the ZnS/ZnO composites were ascribed to the formation of heterojunction between ZnS and ZnO.

## 2. Results and Discussion

### 2.1. Structure and Morphology

**Figure 1:**
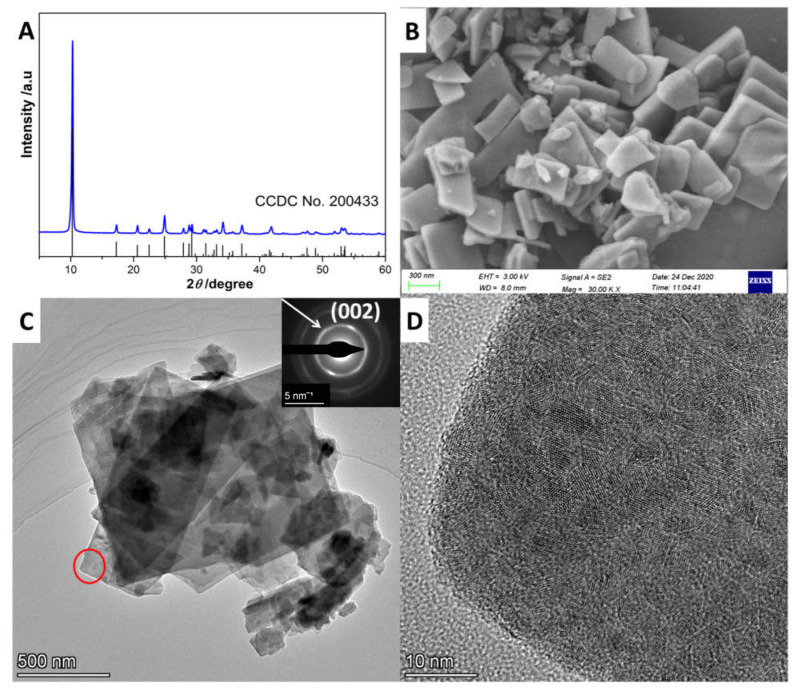
(**A**) XRD diffraction patterns, (**B**) SEM image, (**C**) TEM image and (**D**) HRTEM image of the as-prepared ZnS(en)_0.5_. Inset in C is the SAED image.

The X-ray diffraction (XRD) diffraction pattern of the as-prepared precursor is shown in Figure 1A. As shown, the pattern perfectly matched the referenced date of ZnS(en)_0.5_ (CCDC No. 200433), indicating that ZnS(en)_0.5_ was synthesized successfully [21]. The strongest peak, at about 10°, was assigned to the stacking direction (a axis) of a S-Zn-S layer slab. The scanning electron microscopy (SEM) image in Figure 1B and transmission electron microscopy image (TEM) in Figure 1C show that ZnS(en)_0.5_ had a slab morphology with various lateral sizes and thicknesses, consistent with the crystal structure of ZnS(en)_0.5_. Meanwhile, the selected area electron diffraction (SAED) image (inset in Figure 1C) revealed that ZnS(en)_0.5_ had a polycrystalline structure. The first diffraction ring in the SAED image had a radius of around 3.22 nm, which was ascribed to the diffraction of (002) plane of ZnS(en)_0.5_. Moreover, the high-resolution TEM (HRTEM) image in Figure 1D further confirmed that the bulk ZnS(en)_0.5_ consisted of a nanocrystal of ZnS(en)_0.5_ [7]. 

**Figure 2:**
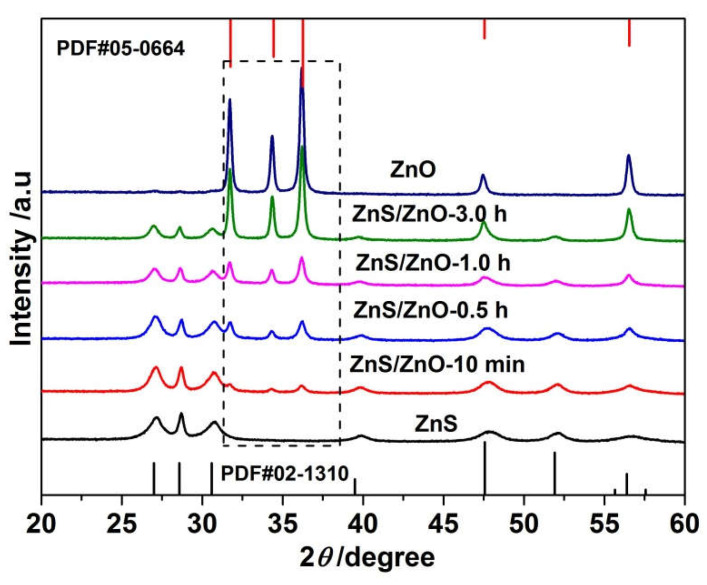
XRD diffractions patterns of the samples obtained via calcination of ZnS(en)_0.5_ at 500 °C for different times including 5 min, 10 min, 0.5 h, 1.0 h, 3.0 h and 5.0 h.

Figure 2 shows the XRD patterns of the samples obtained via calcination of ZnS(en)_0.5_ at 500 °C for different times. When time was 5 min, the obtained sample, defined as ZnS, was assigned to pure hexagonal ZnS. The diffraction peaks at 27.0°, 28.6°, 30.6°, 39.5°, 47.6°, 51.9°, 56.4° were attributed to the (100), (002), (101), (102), (110), (103) and (112) planes of hexagonal ZnS (PDF#02-1310), respectively. Alongside increasing to 10 min, the XRD diffraction patterns of the sample (ZnS/ZnO-10 min) exhibited three extra weak diffraction peaks at 31.7°, 34.3° and 36.2°, which corresponded to the (100), (002) and (101) planes of hexagonal ZnO (PDF#05-0664), respectively, indicating the beginning of the transformation from ZnS to ZnO. With a further increase in the heat treatment time, the intensity of diffraction peaks assigned to ZnS and ZnO tended to decay and enhance, respectively. Until the time extended to 5 h, the obtained sample was pure hexagonal ZnO. 

**Figure 3:**
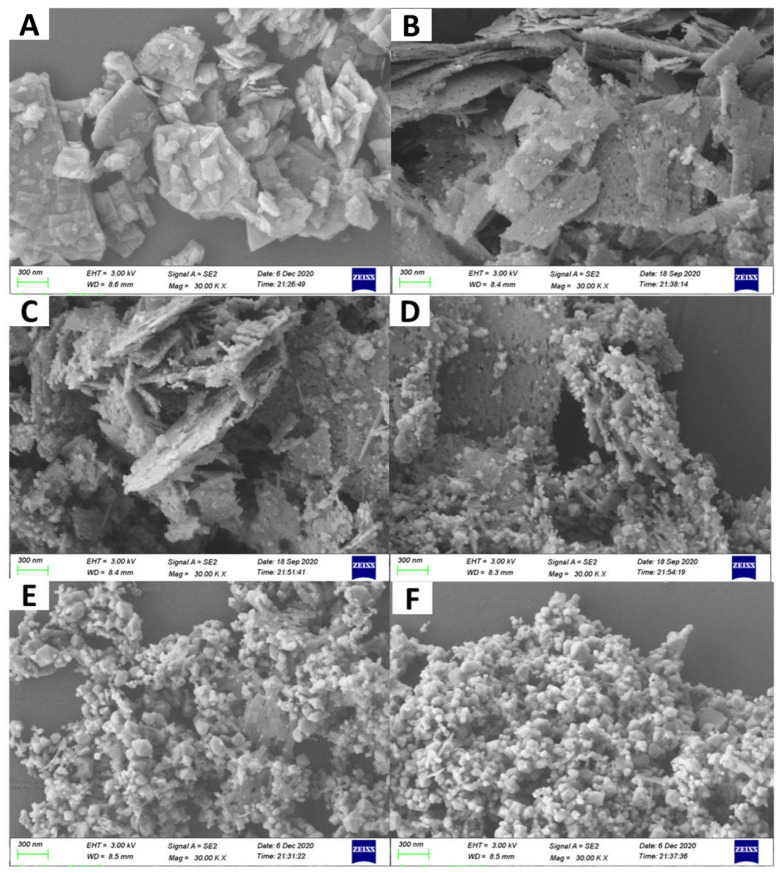
SEM images of the as-prepared samples, (**A**) ZnS, (**B**) ZnS/ZnO-10 min, (**C**) ZnS/ZnO-0.5 h, (**D**) ZnS/ZnO-1 h, (**E**) ZnS/ZnO-3 h, (**F**) ZnO.

**Figure 4:**
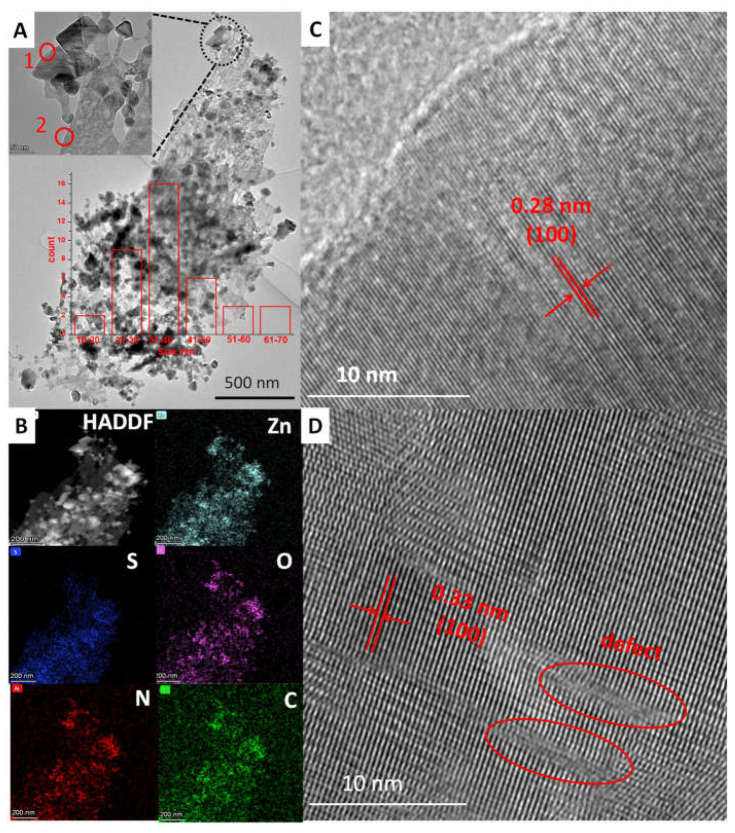
(**A**) TEM, (**B**) HAADF-STEM and Mapping images of ZnS/ZnO-0.5 h, (**C**,**D**) HRTEM images in site 1 and site 2 in A, respectively. Inset in A is the amount of the nanoparticles in different sizes.

The morphologies of the samples were obtained via SEM observation (Figure 3). As shown in Figure 3A, ZnS had a plate-like morphology. Meanwhile, some tiny nanoparticles and pores in nanoscale were observed on ZnS nanoplates for ZnS/ZnO-10 min (Figure 3B). As confirmed by XRD analysis, these nanoparticles should be ZnO. Furthermore, along with the increasing time of heat treatment for the sample of ZnS/ZnO-30 min (Figure 3C), ZnS/ZnO-1 h (Figure 3D) and ZnS/ZnO-3 h (Figure 3E), the amount and the size of the ZnO nanoparticles on the ZnS surface increased and grew up. As for the sample of ZnO (Figure 3F), only nanoparticles with several tens of nanometer were seen. Furthermore, based on the XRD diffraction dates at 2θ = 36.20 (Figure 2), the ZnO particle sizes in ZnS/ZnO compositions were calculated via the Debye–Scherrer Formula of D = Kγ/Bcosθ. The obtained ZnO particle sizes in the samples of ZnS/ZnO-10 min, ZnS/ZnO-0.5 h, ZnS/ZnO-1 h, ZnS/ZnO-3h and ZnO were 13.9, 16.4, 18.1, 23.2 and 24.3 nm, respectively. The morphology and structure of ZnS/ZnO composition, and ZnS/ZnO-0.5 h as a representative sample, was further confirmed by TEM, HAADF-STEM mapping and HRTEM, (shown in Figure 4. TEM image in Figure 4A demonstrated that ZnS/ZnO-0.5 h had a nanoporous plate-like morphology with nanoparticles anchored on. The inset in Figure 4A shows that the surface nanoparticle had a size mainly ranging from 10–70 nm. The HAADFSTEM and mapping images in Figure 4B verified that Zn, S, O, C and N were uniformly dispersed on ZnS/ZnO-0.5 h. The elements of C and N should originate from the residual carbon and the N-doping in composition, because of the decomposition of ethanediamine (en) in the precursor of ZnS(en)_0.5_ under calcination [7]. The existence of O might indicate that partial ZnS was transformed into ZnO. Figure 4C is the HRTEM image of the surface nanoparticle in site 1 in Figure 3A. It shows the nanoparticle-owned clear lattice fringes with a distance of 0.28 nm, which was assigned to the (100) plane of ZnO [22]. It further confirmed ZnS that was transformed into ZnO. Figure 4D shows the HRTEM image of the nanoplate’s counterpart in site 2 in Figure 4A. As shown, it also exhibited distinct lattice fringes with a distance of 0.33 nm, corresponding to the (100) plane of hexagonal ZnS [23]. Additionally, some defects were found, possibly arising from the N-doping, thus leading to the local disorder of ZnS. 

### 2.2. Analysis of Components, BET Surface Area and Energy Band Structure

**Figure 5:**
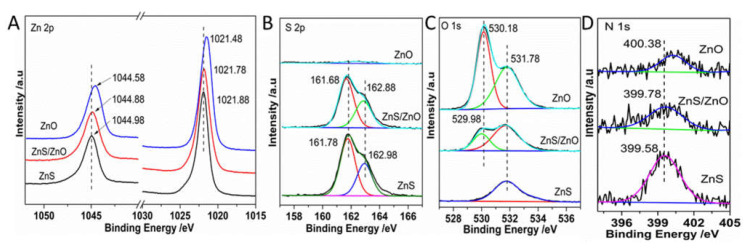
The XPS spectra for the samples. (**A**) Zn 2p, (**B**) S 2p, (**C**) O 1s and (**D**) N 1s. ZnS/ZnO represented the sample of ZnS/ZnO-0.5 h.

To further confirm the surface elemental components and valence states of samples, X-ray photoemission spectroscopy (XPS) was carried out. As shown in Figure 5A, the Zn 2p_3/2_ and Zn 2p_1/2_ of ZnO, ZnS/ZnO-0.5 h, and ZnS located at 1021.48 eV and 1044.58 eV, 1021.78 eV, 1044.88 eV, 1021.88 eV, and 1044.98 eV, respectively, corresponding to the binding energy of Zn^2+^ [24]. The XPS spectra of S^2−^ 2p (Figure 5B) showed that the binding energy of S 2p_3/2_ and 2p_1/2_ for ZnS and ZnS/ZnO-0.5 h were around 161.78 eV and 162.98 eV. No signal of S 2p was detected in ZnO, demonstrating that ZnS was completely transformed into ZnO, thus matching the analysis results of the XRD diffraction. Figure 5C shows O 1s spectra. The binding energies of O 1s, located at about 530.18 eV and 531.78 eV, were assigned to O^2−^ of Zn-O and surface O-H [16], respectively. Moreover, the binding energy of O 1s of Zn-O (529.98 eV) in ZnS/ZnO reduced by 0.2 eV, compared with that of Zn-O in pure ZnO. The negative shift of binding energy of O 1s in ZnS/ZnO was attributed to the formation of a heterojunction between ZnS and ZnO [19], which resulted in a transfer of electron density from ZnS to ZnO, thus leading to an enhanced electron density of the ZnO surface [25]. Furthermore, as shown in Figure 5B and C, during the transformation of ZnS→ZnO, the content of O and S was increased and decreased, respectively. Based on the calculation of the peak area of S 2p of Zn-S and O 1s of Zn-O, the ratio of S/O was 11.3, meaning that the weight percentage of ZnO in the sample of ZnS/ZnO-0.5 h was about 6.9 wt%. Furthermore, Figure 5D shows that ZnS, ZnS/ZnO, and ZnO all exhibited the N 1s spectrum located at 399.58 eV, 399.78 eV and 400.38 eV, respectively, due to N-doping, which was the origination of the visible-light photocatalytic activity of these photocatalysts. Noticeably, the binding energy of N 1s gradually had a positive shift. In terms of ZnS, the N 1s arose from the Zn-N bond. During ZnS→ZnO, the oxidation of Zn-N resulted in the increasing binding energy of N, benefiting the generation of ZnO derived from ZnS directly. 

**Figure 6:**
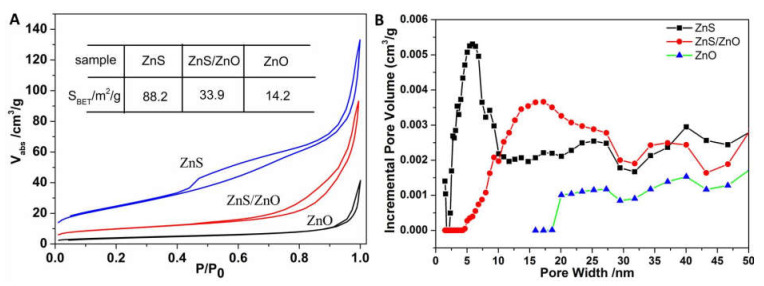
(**A**) BET absorption at different P/P_0_ and (**B**) pore size distribution curves of ZnS, ZnS/ZnO and ZnO. ZnS/ZnO represented the sample of ZnS/ZnO-0.5 h.

Figure 6 shows the specific surface areas and pore size distributions of ZnS, ZnS/ZnO-0.5 h and ZnO, respectively, which were obtained via Brunauer–Emmett–Tell (BET) N_2_ adsorption–desorption isothermal measurements. As shown in Figure 6A, the three samples all had a typical IV adsorption isotherm, proving the existence of mesopores. The BET surface areas were 88.2, 33.9 and 14.2 m^2^/g for ZnS, ZnS/ZnO-0.5 h and ZnO, respectively. Along with the transformation from ZnS to ZnO, a decrease in surface areas was noticed, which should arise from the destruction of the nanoporous plate-like structure of ZnS and the block effect of newborn ZnO nanoparticles. The pore size distribution curves in Figure 6B show that the average pore sizes and the pore volumes were enlarged and decreased, respectively, which further confirmed the collapse of the mesoporous structure, consistent with the SEM and TEM observations discussed above. 

**Figure 7:**
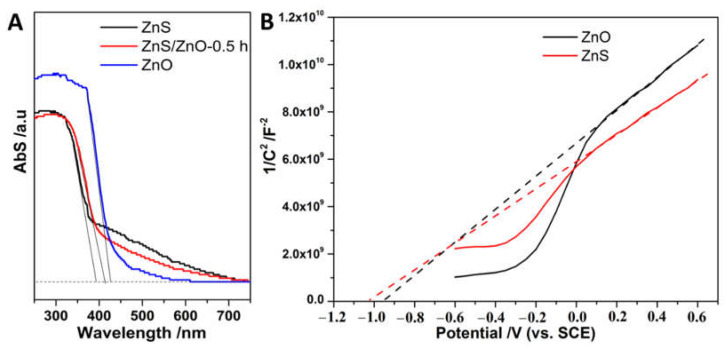
(**A**) The Uv-vis Abs spectra of ZnS, ZnS/ZnO-0.5 h and ZnO; (**B**) Mott–Schottky plots of ZnO and ZnS.

Figure 7A shows the optical absorption property of the samples. As shown, ZnS, ZnS/ZnO-0.5 h and ZnO displayed a band-edge absorption (λ_abs_) around 398, 412 and 427 nm, corresponding to the band gap (E_g_) of 3.16, 3.01 and 2.90 eV estimated via the empirical equation of 1240/λ_abs_ [26], respectively. Noticeably, the three samples had an obvious tailing absorption in the visible region (400–700 nm), indicating that the samples were visible-light response photocatalysts. However, with the transformation from ZnS to ZnO, the samples displayed a decay of spectral absorption in the visible region, which was attributed to the decreased content of doping N atoms. Furthermore, the CB edge position of ZnS and ZnO was evaluated by a Mott–Schottky plots test (Figure 7B). As shown, the conduction band edge (E_CB_) of ZnS and ZnO located at −1.01 V and −0.94 V (vs. SCE, pH = 7), respectively, were higher than the H_2_ evolution potential (−0.66 V, vs. SCE, pH = 7), meaning that the photocatalysts were powerful enough for the photocatalytic reduction of H_2_O for H_2_ evolution. Additionally, based on E_g_ = E_VB_ − E_CB_ [27], the valence band edge (E_VB_) of ZnS and ZnO is located at the position of 2.15 V and 1.96 V. Based on the analysis of energy band edge, the composition of ZnS/ZnO-0.5 h might form the type I heterojunction [8], which could promote the separation of a photogenerated carrier.

### 2.3. Photocatalytic Activity and Stability

**Figure 8:**
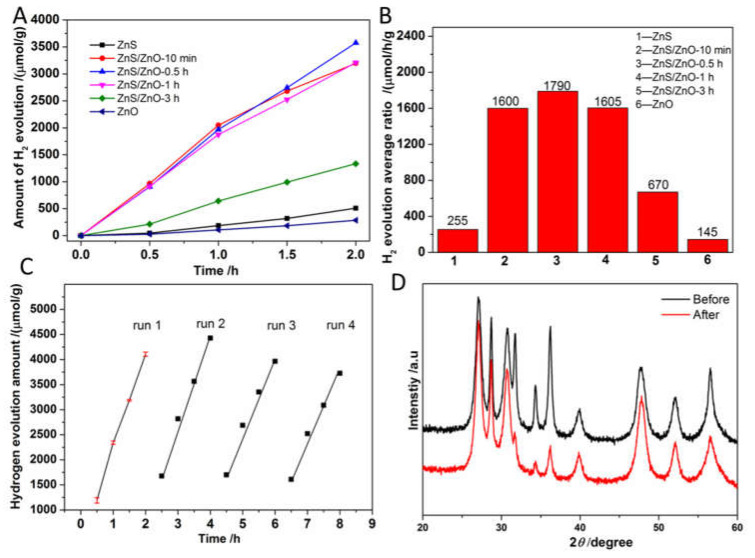
(**A**) and (**B**) Time course of H_2_ evolution activity and the corresponding average rates of H_2_ evolution over the as-prepared samples, (**C**) recycle tests for photocatalytic reaction over ZnS/ZnO-0.5 h, (**D**) the XRD diffraction patterns before and after photocatalytic reaction.

Figure 8A,B shows the time course of H_2_ evolution activity and the corresponding H_2_ evolution rates of the as-prepared samples. As shown, the hydrogen evolution activity exhibited a volcano-like variation with the increasing content of ZnO in photocatalysts. The average rates of H_2_ evolution for pure ZnS and ZnO were only 255 and 145 μmol/h/g, far lower than that of the ZnS/ZnO composites, 1600, 1790, 1605 and 670 μmol/h/g for ZnS/ZnO-10 min, ZnS/ZnO-0.5 h, ZnS/ZnO-1 h and ZnS/ZnO-3 h, respectively. These results demonstrated that ZnS/ZnO composites had advantages in photocatalytic H_2_ evolution, compared with single ZnS or ZnO, and should be ascribed to an efficient separation of photogenerated carriers because of the formation of a heterojunction and type I energy band edge alignment between a ZnS and ZnO component. Figure 8C shows the stability of photocatalytic hydrogen evolution over ZnS/ZnO-0.5 h. As shown, the hydrogen evolution activity decreased by about 15% after 4 recycle tests. The decay of activity was ascribed to the destruction of the interface structure of the photocatalyst, which was confirmed by the XRD diffraction patterns of ZnS/ZnO-0.5 h after recycle photocatalytic tests. As shown in Figure 8D, compared with the XRD diffraction patterns of ZnS/ZnO-0.5 h before the reaction, the diffraction peaks at 31.7°, 34.3° and 36.2° assigned to the diffraction peaks of ZnO became weaker, because ZnO was transformed into ZnS under the condition of SO_3_^2−^/S^2−^ as the sacrificial reagent. With the transformation of ZnO to ZnS, the heterostructure of ZnS/ZnO suffered from a damage, leading to the decay of photocatalytic hydrogen evolution activity, which further demonstrated the importance of the heterojunction for separation of carrier and enhanced photocatalytic activity. Moreover, as shown in Table 1, the N-doped ZnS/ZnO heterojunction photocatalyst in this work displayed a competitive activity for H_2_ evolution under visible light, as compared with the reported ZnS/ZnO compositions.

## 3. Materials and Methods

### 3.1. Materials

The H_2_PtCl_6_·_6_H_2_O, (A. R., Sinopharm Chemical Reagent Co. (SCRC, Shanghai, China), ethanol (EtOH, A. R., SCRC), deionized water (home-made), ethanediamine (en, A. R., SCRC), thiourea (A. R., SCRC), ZnCl_2_ (A. R., SCRC.). N,N-dimethylformamide (DMF, A. R., SCRC), Na_2_S·H2O and Na_2_SO_3_ (A. R., SCRC.).

### 3.2. Preparation of Photocatalysts 

Synthesis of ZnS(en)_0.5_: 0.191 g of ZnCl_2_, 0.64 g of thiourea and 50 mL of ethanediamine were added into a 100 mL teflon-lined autoclave, and the mixture was stirred for 1 h. Subsequently, the autoclave was sealed and maintained at 160 °C for 12 h and naturally air cooled. The resulting white solid products were centrifuged, washed with absolute ethanol and distilled water several times, and then dried at 40 °C overnight. 

Synthesis of ZnS/ZnO composition: The as-prepared 40 mg of ZnS(en)_0.5_ was spread into a combustion boat with a capacity of 5 mL. Then, the sample was rapidly put into a muffle furnace with a temperature of 500 °C for some time (t) and taken out immediately. The samples, calcinated at different times (t = 5 min, 10 min, 0.5 h, 1 h, 3 h and 5 h), were respectively defined as ZnS, ZnS/ZnO-5 min, ZnS/ZnO-10 min, ZnS/ZnO-0.5 h, ZnS/ZnO-1 h, ZnS/ZnO-3h and ZnO.

### 3.3. Characterization 

Structure and Morphology: XRD patterns were recorded on a X’Pert3 Powder (PANalytical, Almelo, Netherlands) X-ray diffractometer with Cu Ka radiation operated at 40 kV and 40 mA. To obtain the transmission electron microscopy (TEM) images, high-resolution (HR) TEM images and STEM-EDX mapping, the samples were dropped on a Mo grid and operated on a Talos F200S (Thermo, Waltham, USA). X-ray photoelectron spectroscopy (XPS) measurements were performed on a ThermoFischer system (Thermo, Waltham, USA) with a monochromatic Al Kα source. XPS dates were calibrated by C1s = 284.8 eV. Ultraviolet-visible absorption spectra was obtained using UV-2600 (Shimadzu, Tyoto, Japan), BaSO4 as the reference. Field-emission scanning electron microscopy (FESEM, Carl Zeiss Sigma 300, Oberkochen, Germany) was used to determine the morphology of the samples. The Brunauer–Emmett–Teller (BET) surface area was measured with an TriStar II Plus apparatus (Micromeritics Instrument Corp, Atlanta, USA).

Electrochemical measurements: The working electrode was prepared on fluorinedoped tin oxide (FTO) glass, which was cleaned by sonication in acetone, ethanol and deionized water for 30 min each. Next, 5 mg of photocatalyst powder was dispersed in 0.5 mL of dimethylformamide (DMF) under sonication for 2 h to produce slurry. Then, 10 μL of the as-prepared slurry was spread onto the conductive surface of the FTO glass to form a photocatalyst film with an area of 0.25 cm^2^. After air drying naturally, the uncoated parts of the electrode were isolated with an epoxy resin. Subsequently, the electrodes were put into an oven at 100 °C for 2 h. For the Mott–Schottky experiment, the potential ranged from −0.6 to 0.6 V with an increase in voltage of 50 mV, and the amplitude was 5 mV under the frequency of 500 Hz. The measurement was also performed in a conventional three electrode cell, using a Pt wire and a SCE electrode as the counter electrode and reference electrode, respectively. The electrolyte was 0.2 M of Na_2_SO_4_ aqueous solution without additive (pH = 6.8).

Photocatalytic tests: The photocatalytic reactions were carried out in a photocatalytic hydrogen evolution system (MC-H20II, Merry Change Co., Beijing, China). For this, 20 mg photocatalyst was suspended in 50 mL of (0.1 M) Na_2_SO_3_/(0.1 M) Na_2_S aqueous solution. Next, 1%Pt was introduced into the reaction system via in situ photodeposition of H_2_PtCl_6_.The suspension was then thoroughly degassed and irradiated with visible light (λ > 400 nm) by using a 300 W Xenon lamp (PLS-SXE300D, Perfectlight Co., Beijing, China). H_2_ was detected at set intervals, automatically, by an online gas chromatograph. For photocatalytic recycle tests over ZnS/ZnO-0.5 h, three parallel photocatalytic H_2_ evolution tests were firstly performed. Then, the photocatalyst was recovered after every parallel test. The recovered photocatalyst was used for next run test.

## 4. Conclusions

N-doped ZnS/ZnO composite with heterostructure was prepared successfully by the in situ transformation of ZnS(en)_0.5_ with heat treatment under air atmosphere. The content of ZnO in ZnS/ZnO was able to be controlled via adjustment of the calcination time. SEM demonstrated ZnO nanoparticles were dispersed on the ZnS surface. TEM further verified that the surface ZnO nanoparticles had sizes ranging from 10 to70 nm and anchored on porous ZnS nanoplate firmly. XPS verified that the N was doped into ZnS/ZnO during the in situ transformation of ZnS(en)_0.5_ to ZnS/ZnO, and a heterojunction was formed between ZnS and ZnO. Photocatalytic hydrogen evolution experiments demonstrated that the sample of ZnS/ZnO-0.5 h with 6.9 wt% of ZnO had the best H_2_ evolution activity (1790 μmol/h/g) under visible light irradiation (λ > 400 nm), about 7.0 and 12.3 times of that of the pure ZnS and ZnO, respectively. The enhanced activities of the ZnS/ZnO composites were ascribed to the intimated hetero-interface between components and efficient transfer of photo-generated electrons from ZnS to ZnO. However, although the N-doped ZnS/ZnO obtained via the in situ transformation method achieved visible-light photocatalytic H_2_ evolution activity, the catalytic stability and absolute H_2_ evolution activity of ZnS/ZnO should be improved further for meeting the potential demand of hydrogen energy. Furthermore, the as-prepared N-doped ZnS/ZnO could be used for the photoanode material of dye sensitized solar cells because of the existence of the heterojunction with staggered conduction band edges and nanoporous structure, which availed the transmission of photoelectrons and absorption of dye molecules, thus improving the photoelectric conversion efficiency.

## Figures and Tables

**Table 1:** Comparison of the photocatalytic H_2_ evolution activities of ZnS/ZnO.

Photocatalyst	Sacrificial Agent in Aqueous Solution	H_2_ Evolution Activity /μmol·g^−1^·h^−1^	Light/nm	Reference
N-doped ZnS/ZnO-Pt%	0.1 M S^2−^/0.1 M SO_3_^2−^	1790	λ > 400	This work
ZnS/ZnO	CH_3_OH 50 % v/v	1242	254	[15]
ZnS/ZnO@CT	5% lactic acid	37.1	400–780	[28]
ZnS@ZnO	0.1 M S^2−^/0.1 M SO_3_^2−^	≒4600	Xenon lamp	[18]
ZnS-ZnO	0.25 M S^2−^/0.35 M SO_3_^2−^	22	λ > 420	[29]
Pt/ZnS-ZnO	0.1 M S^2−^/0.1 M SO_3_^2−^	10,700	Xenon lamp	[19]
ZnS/ZnO	0.45 M S^2−^/0.45 M SO_3_^2−^	374	λ > 400	[30]
ZnS/ZnO	0.1 M S^2−^/0.1 M SO_3_^2−^	≒250	λ > 420	[31]
ZnS-ZnO	0.4 M S^2−^	494.8	Xenon lamp	[32]
Pt/ZnS@ZnO	Water	87.6	Xenon lamp	[33]
ZnO/ZnS	0.1 M S^2−^/0.1 M SO_3_^2−^	415.3	λ > 420	[20]

## Data Availability

The data presented in this study are available on request from the corresponding author.

## Sample Availability

Samples of the compounds are not available from the authors.
